# Biomechanical analysis of stress distribution and failure risk in mandibular incisors restored with resin-bonded fixed partial dentures using CAD/CAM materials and restoration designs

**DOI:** 10.3389/fbioe.2024.1501815

**Published:** 2024-11-21

**Authors:** Hailiang Wang, Jingwen Cai, Jie Liang, Yong Wang, Yunsong Liu

**Affiliations:** ^1^ Fujian Key Laboratory of Oral Diseases, School and Hospital of Stomatology, Fujian Medical University, Fuzhou, China; ^2^ Department of Implantation, Yantai Stomatological Hospital, Binzhou Medical University, Yantai, China; ^3^ Department of Prosthodontics, Peking University Hospital of Stomatology, Beijing, Beijing Municipality, China

**Keywords:** resin-bonded, finite element analysis, dental prosthesis design, dental stress analysis, anterior tooth loss

## Abstract

**Background:**

Computer-aided design/manufacturing (CAD/CAM) materials are widely used in resin-bonded fixed partial dentures (RBFPDs), but their suitability across different designs has not been fully assessed. This study compares the stress distribution and failure probability of mandibular incisors restored by RBFPDs with various CAD/CAM materials.

**Materials and methods:**

Finite-element models of single- and double-ended RBFPDs were created using cone-beam computed tomography (CBCT) data. Five CAD/CAM materials (IPS e.max CAD, IPS e.max ZirCAD, Vita Enamic, Lava Ultimate, Vitablocs MarkII) were tested under vertical and oblique (45°) loading with a 100 N force. Stress distribution and failure risk were evaluated for each material and design.

**Results:**

Oblique loading produced the highest stress and displacement for single-ended RBFPDs. Lava Ultimate had the largest displacement and principal stress, while IPS e.max ZirCAD showed the highest equivalent stress. IPS e.max CAD exhibited the lowest displacement and principal stress among double-ended RBFPDs under oblique loading.

**Conclusion:**

This study demonstrated that double-ended RBFPDs experience lower stress and strain compared to single-ended designs, particularly under oblique loading. Vita Enamic had the highest failure risk, while IPS e.max ZirCAD had the lowest. These insights into stress distribution and material performance offer valuable guidance for material selection and restoration design, aiming to improve the longevity and success of RBFPDs in mandibular incisor restorations.

## Introduction

Mandibular anterior tooth loss is commonly caused by caries, periodontitis, and trauma, with an incidence rate reaching up to 0.2%–15.7% (Schneider-Moser et al., 2024). Given that anterior teeth play a critical role in facial aesthetics and harmony, restoring missing anterior teeth is of paramount importance. Due to the unique anatomical structure of the lower anterior teeth, implant restoration is often limited by factors such as thin gingival biotypes, insufficient alveolar bone thickness, and narrow interdental spaces. Moreover, the use of traditional fixed dental bridges may result in unnecessary loss of healthy tooth structure from the adjacent teeth. In such cases, resin-bonded fixed partial dentures (RBFPDs) have become a widely adopted restorative solution (Kern and Sasse, 2011a; Bilir et al., 2022). The dual-wing design of RBFPDs not only enhances retention but also minimizes harm to adjacent teeth, exhibiting excellent mechanical performance, particularly in anterior teeth where occlusal forces are relatively low (Zhang et al., 2020; Miettinen and Millar, 2013).

By the late 20th century, RBFPDs began to be fabricated from all-ceramic materials, overcoming the aesthetic and mechanical limitations of metal-ceramic and fiber-reinforced composite resin fixed partial dentures. With the rapid advancement of digital dentistry and computer-aided design/manufacturing (CAD/CAM) materials, many chairside numerical controls (NC) milling materials have been used in RBFPD fabrication. Common computer numerical control (CNC)-machinable ceramic materials include resin matrix composite blocks, ceramic matrix resin-infiltrated composite blocks, feldspar-reinforced glass ceramic blocks, lithium disilicate glass ceramic blocks, and zirconia ceramic blocks (Calheiros-Lobo et al., 2022; Skorulska et al., 2021). While the mechanical properties of these materials are generally suitable for RBFPDs, their physical properties—such as strength, toughness, and elastic modulus—can directly impact the retention and stability of the restorations (Shaikh et al., 2022; Yin et al., 2024).

Resin matrix composite blocks and ceramic matrix resin-infiltrated composite blocks are known for their high toughness and impact resistance, making them ideal for anterior tooth restorations. However, their relatively poor wear resistance may limit their use in regions subjected to high occlusal forces. Feldspar-reinforced glass ceramic blocks, noted for their superior aesthetics, are suitable for anterior restorations with high aesthetic demands. Nonetheless, their brittleness makes them prone to fracture under high stress. Lithium disilicate glass ceramic blocks offer a combination of high strength and aesthetics, making them suitable for restorations subjected to higher occlusal forces, though they require more precise equipment for fabrication due to their challenging processing. Zirconia ceramic blocks, with their exceptional strength and toughness, are considered ideal for posterior restorations. However, their lower translucency may limit their aesthetic appeal in anterior restorations (Silva et al., 2023; Duarte et al., 2016; Mörmann et al., 2013).

Miettinen and Millar (2013) reviewed several clinical follow-up studies on the clinical performance of all-ceramic RBFPDs, with particular attention to their long-term stability. Their findings highlighted that bridge fractures and debonding were the primary causes of failure, which were closely linked to the type of all-ceramic material used. Different materials exhibited varying responses to the complex mechanical stresses within the oral cavity, with some materials more susceptible to stress concentration, leading to fractures or debonding. Depending on the number of retainers, all-ceramic RBFPDs are generally classified into double-retainer (DE) and single-retainer (SE) designs. Rosentritt et al. (2009a) *in vitro* tests demonstrated that DE RBFPDs showed superior biomechanical strength and stability compared to SE RBFPDs. This is likely because DE designs more effectively distribute occlusal forces, reducing stress concentration and enhancing durability.

However, Koutayas et al. (2002) provided a different perspective. Using a biaxial chewing simulation model to more accurately replicate the oral stress environment, they found that SE RBFPDs performed better in certain scenarios. This could be due to the reduced load on the retainer and minimized stress at the bonding interface in SE designs. Thus, in specific clinical situations, SE designs may offer a more appropriate solution.

While various CAD/CAM materials are suitable for RBFPD fabrication, differences in their physical properties—such as strength, toughness, and wear resistance—can significantly affect the clinical outcomes of the restorations. For instance, insufficient wear resistance may lead to premature degradation, while inadequate strength may increase the risk of bridge fracture. Therefore, the clinical performance of these materials varies under different conditions, necessitating careful selection based on individual patient needs. To systematically evaluate the suitability of different materials in both SE and DE designs, this study employs three-dimensional finite element analysis (FEA) and Weibull analysis to explore their stress distribution and failure risks. The objective of this study is to address the aforementioned scientific questions and provide reliable clinical guidance.

## Materials and methods

### Data source

A 30-year-old male patient was selected for cone-beam computed tomography (CBCT) data collection. The patient had complete dentition, normal tooth arrangement, a normal occlusal relationship, healthy periodontal tissue, and normal crown anatomy. The patient provided written informed consent.

### Establishment of 3D model

**FIGURE 1:**
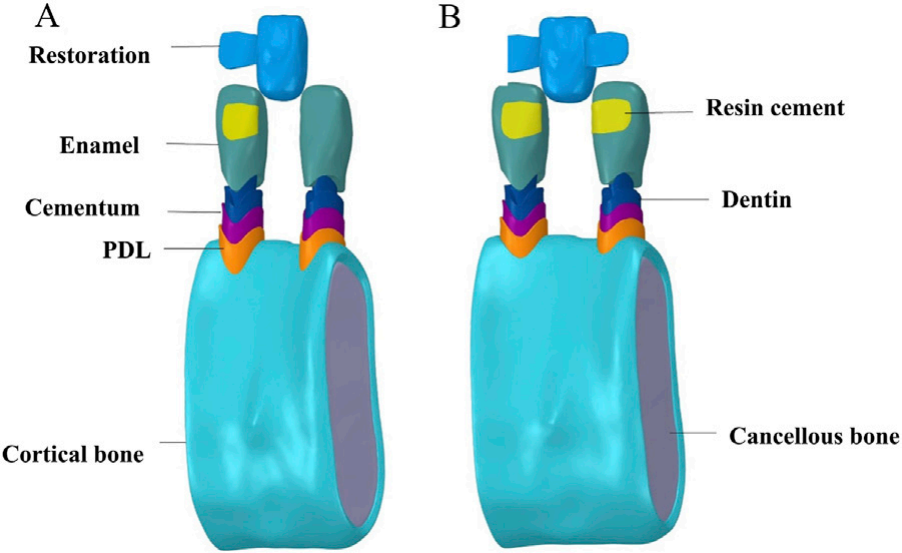
Material components of simulation models. **(A)** single-ended resin-bonded fixed partial denture. **(B)** double ended resin-bonded fixed partial denture.

This study aimed to establish a 3D finite-element model of the left lower central incisor with SE or DE RBFPDs made of different CAD/CAM materials, with the left and right lower central incisor as abutments. The Digital Imaging and Communications in Medicine (DICOM) data of the mandibular image of the patient were obtained by CBCT and directly imported into Mimics 21.0 software for modeling. The curved surface models of the left lower incisor, right lower central incisor, and mandible were extracted and imported into Geomagic Studio 2015 software to smooth and trim the model. The enamel model of 0.6 mm, the cementum model with a thickness of 0.2 mm, and the dentin model were then created using the shelling function. The periodontal ligament model with a thickness of 0.25 mm was produced by exfoliating and thickening the tooth’s root. In addition, the adhesive layer of 0.1 mm and the wing plate of 0.5 mm were constructed based on the surface topology of the crown’s lingual side. A cortical bone model with a thickness of 1 mm, including the 1 mm thick bone plate surrounding the tooth root, was generated along with a cancellous bone model enclosed within it, based on the segmental model of the mandible. This generates a missing model of the left lower central incisor, ensuring that the objects are in close contact with each other, with no defects. The 3D restoration design software was imported into the 3Shape Dental System 2020 software to design the bridge part of the adhesive bridge, with the bridge body specifically designed as a saddle-less (suspended) type to minimize mechanical interference from the gingiva. After the bridge design was completed, it was imported to Geomagic Studio software together with the wing plate to generate a complete SE or DE RBFPD model, ensuring consistency in the structural analysis. All models were optimized to fit the surface accurately and were imported into the FEA pre-processing software SpaceClaim (Ansys), as shown in Figure 1. The 3D solid model in SCDOC format was imported into the FEA software, and two basic finite-element models were developed, as shown in Table 1.

**TABLE 1:** Mechanical properties of materials used in finite element analysis and Weibull analysis.

Materials	Young’s modulus (MPa)	Poisson’s ratio	Characteristic strength (MPa)	Weibull modulus(m)
Enamel (Lin et al., 2009; Magne et al., 2012)	84,100	0.30	42.41	5.53
Dentin (Lin et al., 2009; Magne et al., 2012)	18,600	0.27		
Cementum (Malek et al., 2003; Srivicharnkul et al., 2005)	2,398	0.30		
PDL (Malek et al., 2003; Srivicharnkul et al., 2005)	68.9	0.45		
Cancellous bone (Malek et al., 2003; Srivicharnkul et al., 2005)	1,370	0.30		
Cortical bone (Malek et al., 2003; Srivicharnkul et al., 2005)	13,700	0.30		
Resin cement (Lin et al., 2009; Dejak and Mlotkowski, 2008)	8,300	0.35	453.80	4.02
IPS e.max CAD (Wendler et al., 2017; Belli et al., 2017)	102,700	0.35	609.80	13.40
IPS e.max ZirCAD (Wendler et al., 2017; Belli et al., 2017)	204,100	0.25	1,303.21	12.30
Vita Enamic (Wendler et al., 2017; Belli et al., 2017)	37,800	0.24	193.45	18.80
Lava Ultimate (Wendler et al., 2017; Belli et al., 2017)	12,700	0.45	300.64	10.90
Vitablocs MarkII (Wendler et al., 2017; Belli et al., 2017)	71,300	0.23	118.65	19.90

### Mechanical parameters

This model assumes that all components are homogeneous and isotropic linear elastic materials with small deformations. The model’s interfaces remained stable under stress and did not slide with each other (no overlap or splitting). Five CAD/CAM materials were used to fabricate the RBFPDs. The elastic moduli and Poisson’s ratios of various structural materials were obtained from the literature and manufacturers within the past 5 years.

### Finite-element model parameter setting

#### Contact relationship between materials

The contact relationship between the components in close contact is defined as the binding constraint.

#### Loading and analysis of the force

The occlusal mode of normal people when chewing food was simulated. The loading position was the incisor end of each tooth, directionally axial, and 45° from the labial side. The magnitude of the loading force was 100 N, which is similar to the magnitude and direction of chewing force of normal lower anterior teeth during mastication (Ferrario et al., 2004).

#### Boundary condition

The boundary condition was fixed at the bottom of the cortical bone to ensure that the displacement of six degrees of freedom was zero.

#### 3D finite-element meshing

In this study, the free meshing method of Ansys 2020 R2 software (Ansys) was used to mesh all objects, including enamel, resin cement, restoration related to stress concentration, and tetrahedral element type. Simultaneously, the grid convergence test (with a convergence tolerance of 20%) was performed to ensure that the meshing did not significantly interfere with the results (Dal Piva et al., 2017; Tribst et al., 2018).

#### 3D FEA

The Ansys 2020 R2 software was used to solve the defined 3D finite-element model and output the displacement and node stress. The stress value (equivalent stress), maximum principal stress, and maximum displacement were analyzed.

#### Weibull analyses

All materials in our analyses were assumed to be linear elastic and their failure was assumed to follow the normal stress failure criterion in Weibull risk-of-rupture analysis (Sato et al., 1995; Kelly et al., 1995). Failures were presumed to occur from the highest principal tensile stress on stress concentration areas. Therefore, the survival probability in Weibull analysis, $P_{S}$ , was calculated as follows:

$$P_{S}\left(\sigma \right)=e^{-{\left(\frac{\sigma }{\sigma_{0}}\right)}^{m}}$$

where $P_{S}$ represents the survival probability of a node at stress σ (for load F), σ represents the failure stress (maximum principal stress), $\sigma_{0}$ represents the characteristic strength, which is a normalizing parameter corresponding to the stress at which 63.2% of specimens fail, and m represents the Weibull modulus, which is a material parameter determined by the flaw size distribution (Kelly et al., 1995; Gonzaga and Cesar, 2011). When loaded, a restoration will survive until the risk-of-rupture reaches a critical value at any one of the multiple failure sources. For a system with n failure risks, the total survival probability $P_{S}$ is the product of n individual survival probabilities (Kelly et al., 1995; Lin et al., 2009):

$$P_{S}=\prod_{i=1}^{n}{P_{S}}_{i}$$

where i is a natural number. In RBFPDs, it was observed that the stress concentration area of the enamel, the cement layer and the joint of the restoration was at risk of failure. Thus, the failure probability, $P_{f}$ , for the total system is determined by:

$$P_{f}=1-{P_{f}}_{e}\times {P_{f}}_{c}\times {P_{f}}_{j}$$

The failure probability and load curves of each RBFPD and whole system (restoration, cement, and enamel) of the lower anterior teeth were calculated and compared (Huang et al., 2020). $\sigma_{0}$ and m of different materials were obtained from the literature (Magne et al., 2012; Malek et al., 2003; Srivicharnkul et al., 2005; Celebi et al., 2017; Dejak and Mlotkowski, 2008; Wendler et al., 2017; Belli et al., 2017) (Table 1).

## Results

**FIGURE 2:**
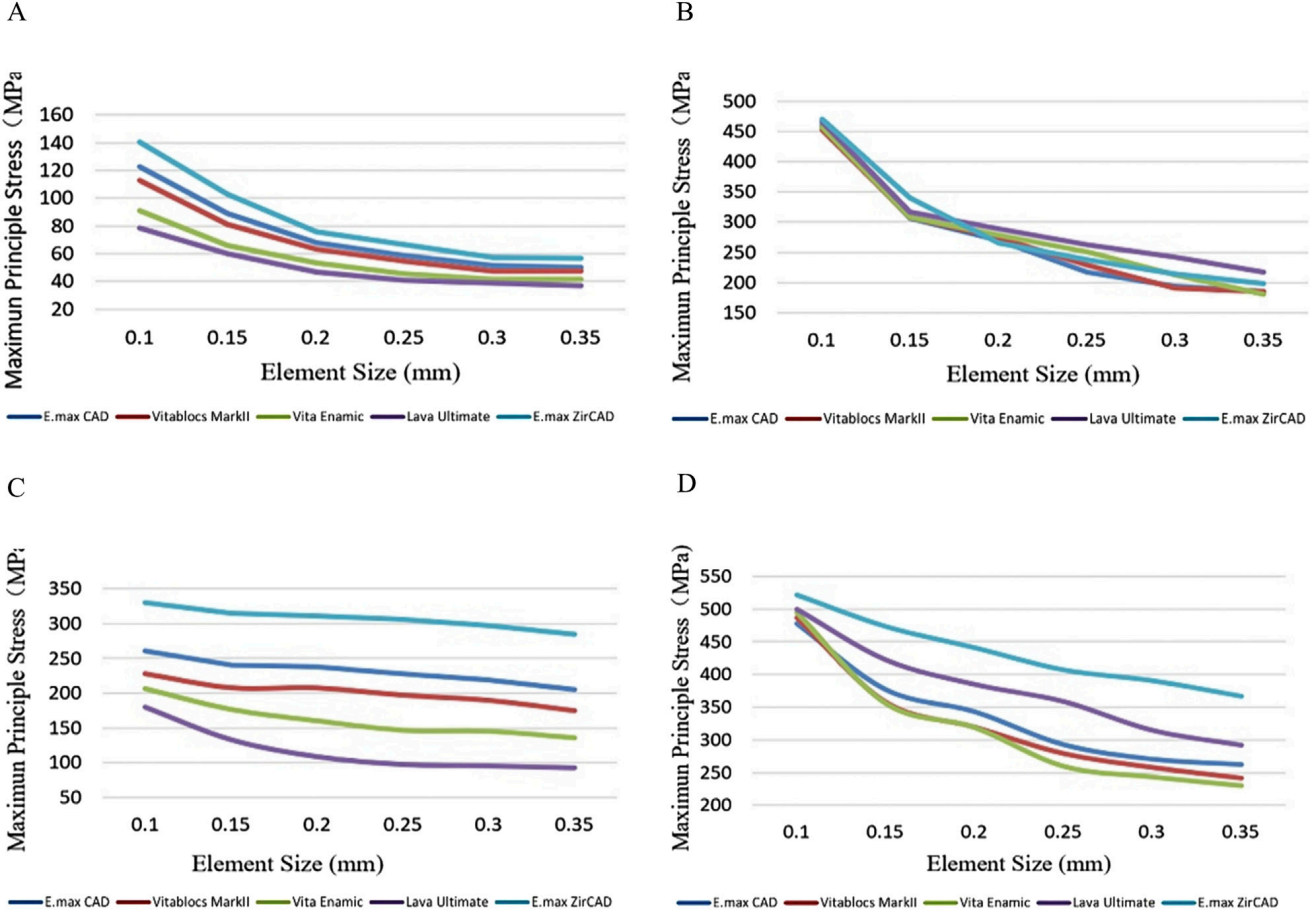
Grid convergence test (20%) for **(A)** double-ended model with vertical load, **(B)** single-ended model with vertical load, **(C)** double-ended model with oblique load, and **(D)** single-ended model with oblique load.

Two basic finite-element models were constructed. Simultaneously, the grid convergence test was carried out for all components, as shown in Figure 2. The maximum principal stress in the key area of the adhesive bridge restoration was stable with the continuous iteration of mesh refinement under vertical and oblique 45°, 100 N loads (Peyron, 2023). These results show that the maximum principal stress is numerically accurate, and all modeling assumptions are valid. The final mesh size was 0.2 mm, and the same mesh size was used in all models considering the computing power of the computer and grid quality (Sato et al., 1995). The element quality coefficient of the grid index of the SE model was 0.82585. After grid division, the final index was 2,881,477 nodes and 1,924,465 units, and the element quality coefficient of the DE model grid index was 0.82534. After dividing the grid, the final index was 2,910,403 nodes and 1,941,248 units.

### Stress and strain of the restoration

**FIGURE 3:**
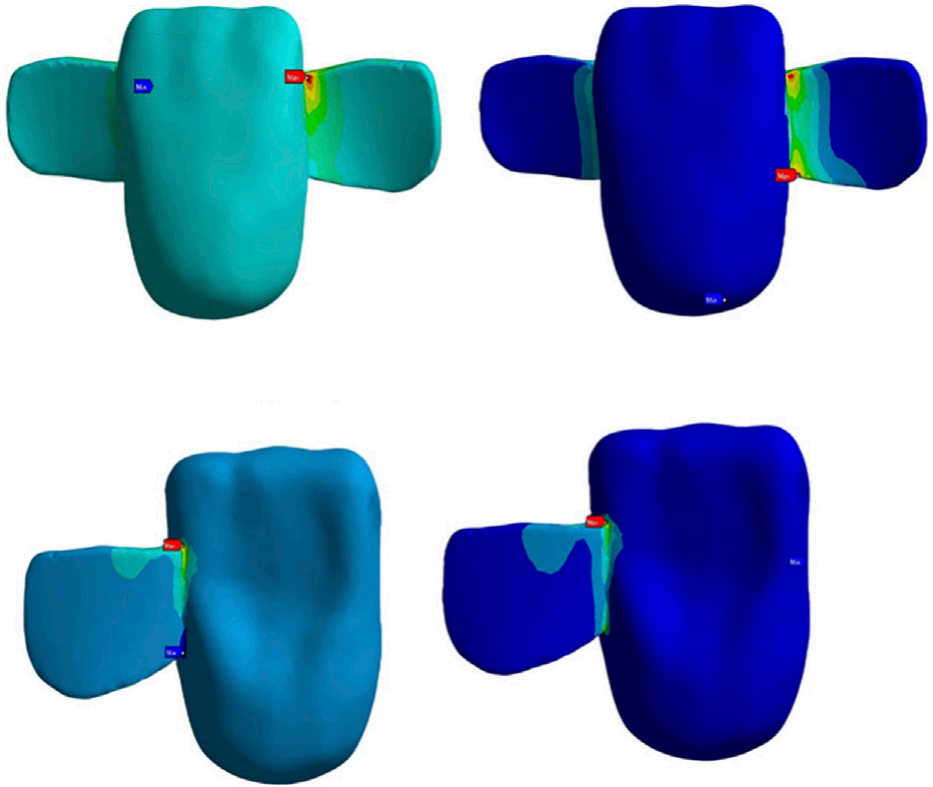
Stress concentration of the restorations: the areas of stress concentration within the resin-bonded fixed partial dentures (RBFPDs). The regions with higher stress are highlighted in red, indicating potential failure points under occlusal load, particularly in the connector and joint areas.

**FIGURE 4:**
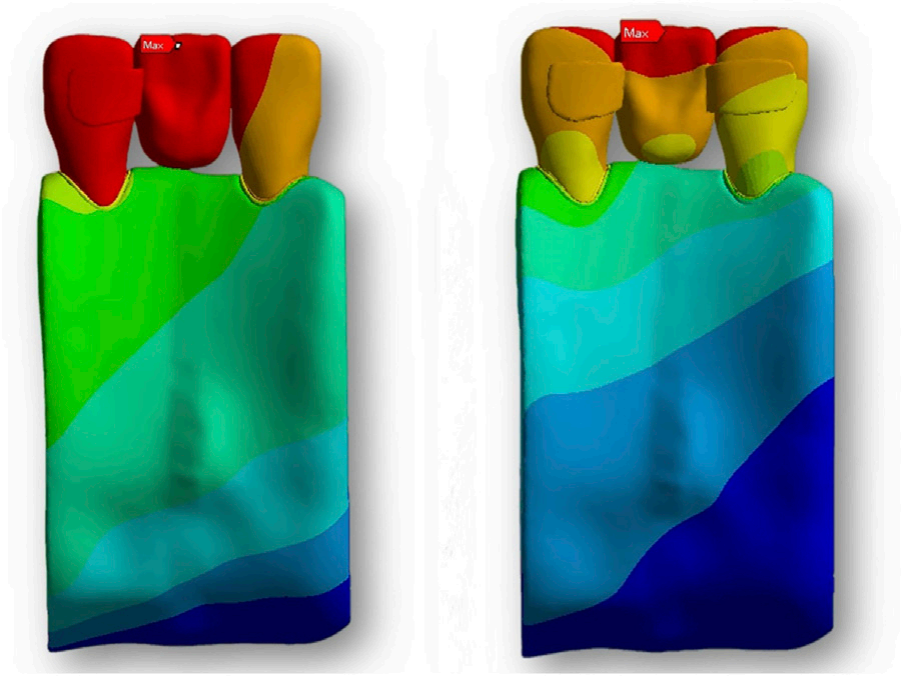
Displacement analysis of RBFPDs under load: the overall displacement of the RBFPDs under vertical and oblique loads. The color gradient illustrates displacement magnitude, with darker colors (especially red) representing areas of maximum displacement, which highlights potential zones of structural weakness or instability.

**FIGURE 5:**
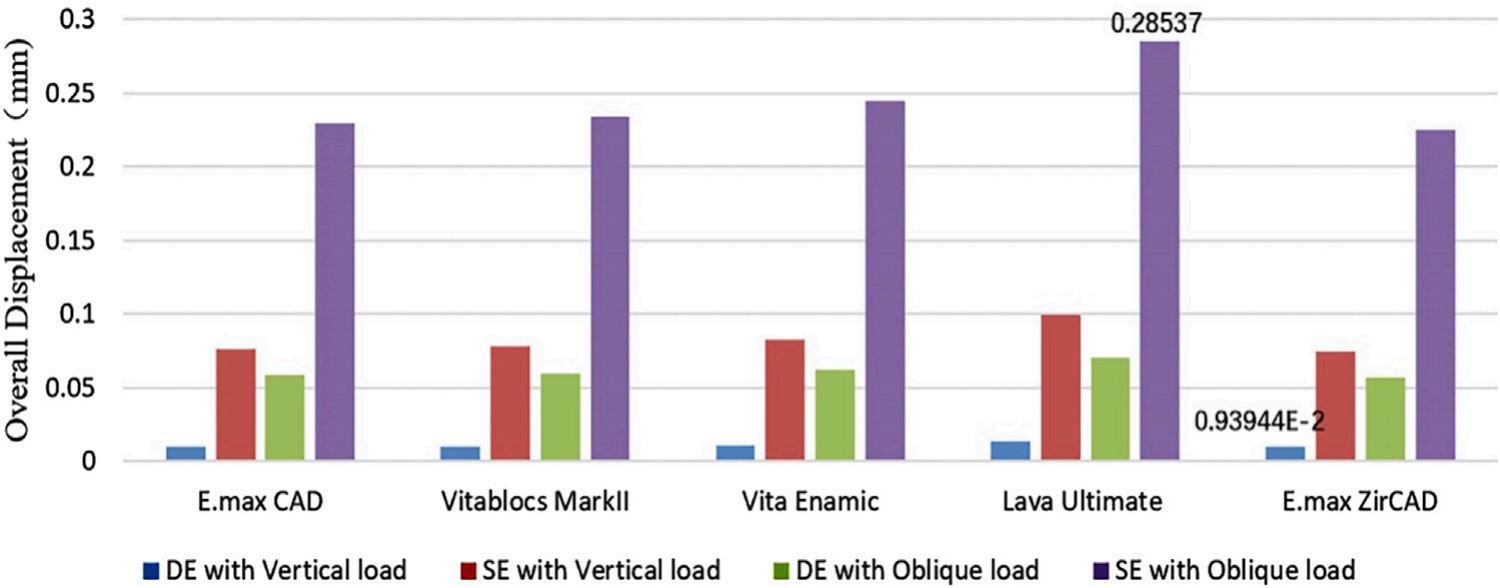
Quantification of the overall displacement of the resin-bonded fixed partial dentures (Blue: DE with Vertical load, Red: SE with Vertical load, Green: DE with Oblique load, and Purple: SE with Oblique load).

**FIGURE 6:**
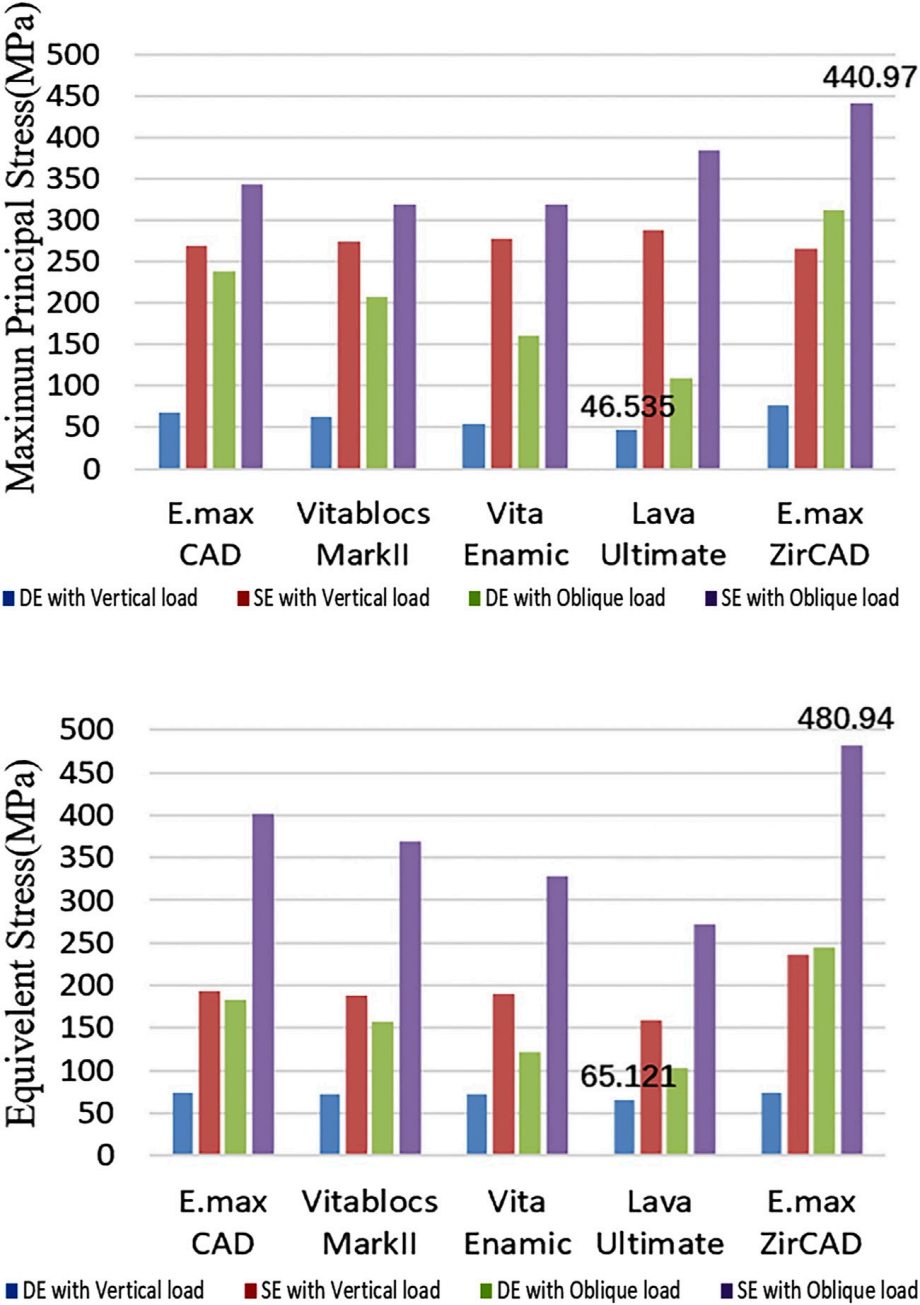
Quantification of the stress of the restorations (Blue: DE with Vertical load, Red: SE with Vertical load, Green: DE with Oblique load, and Purple: SE with Oblique load).

The cloud map shows that the joint of the restoration is the area of stress concentration. The edge is the maximum stress, and the stress of the wing plate decreases gradually from the joint’s side to the far gap side, as shown in Figure 3. The change in displacement indicates that the load value of the RBFPDs bridge is the largest, and gradually decreases towards the wing. Moreover, the overall displacement of the RBFPDs system (restoration, resin cement, and enamel) is consistent, as shown in Figure 4. The displacement of the Lava Ultimate SE RBFPD was the largest among all test groups under oblique loading. The displacement of the IPS e.max ZirCAD DE RBFPD was the smallest when loading vertically, as shown in Figure 5. The displacement of the IPS e.max CAD DE RBFPD under vertical loading was not significantly different from that of IPS e.max ZirCAD. The principal stress value of SE RBFPDs under oblique loading was the largest among all test groups, as shown in Figure 6. Among these, the principal stress value of Lava Ultimate SE RBFPDs is the largest under oblique loading, whereas that of IPS e.max CAD DE RBFPDs was the smallest. The equivalent force value of the IPS e.max ZirCAD SE RBFPDs was the largest under oblique loading. However, that of the Lava Ultimate DE RBFPDs was the smallest when loaded vertically.

### Stress of the resin cement

**FIGURE 7:**
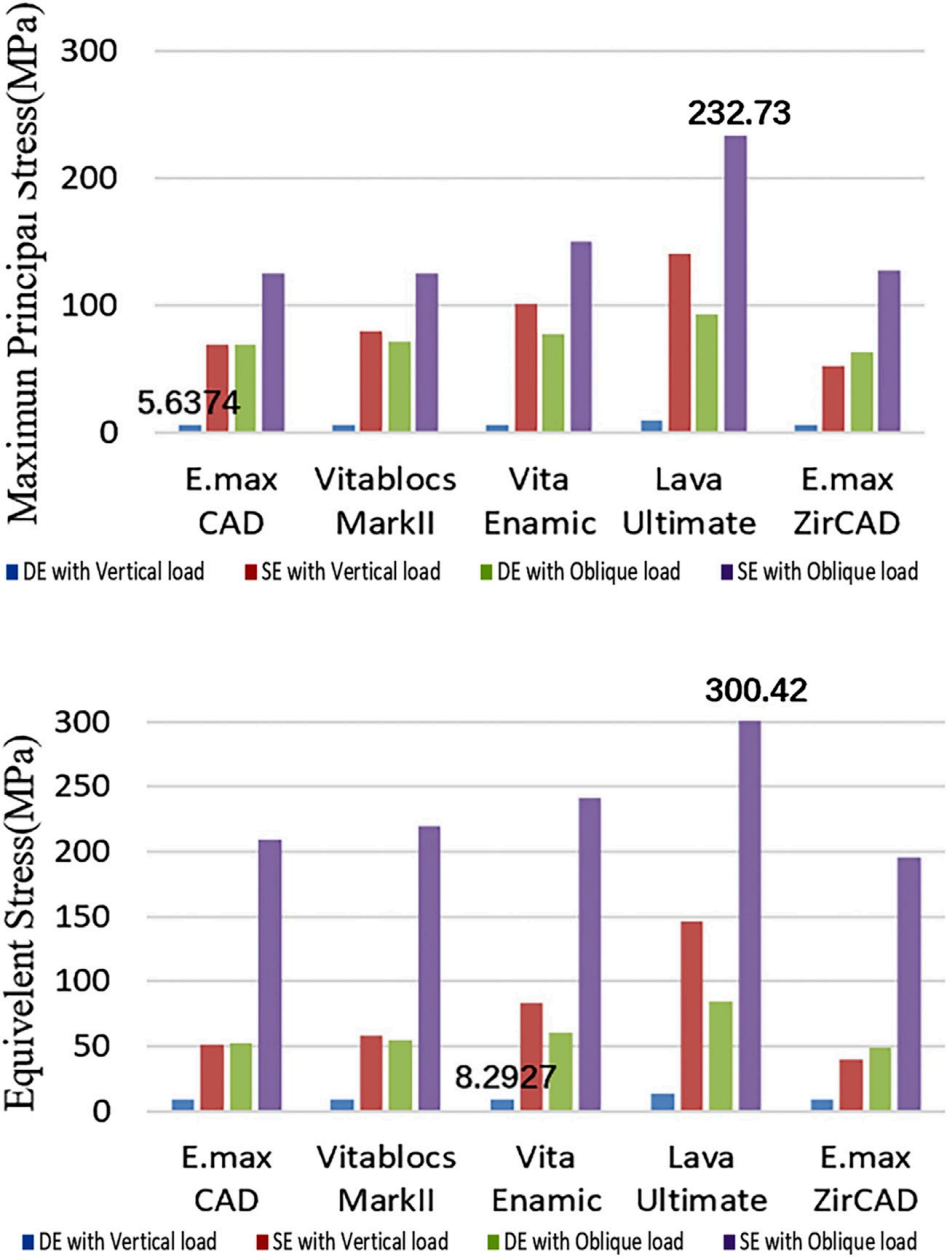
Quantification of the stress or resin cements (Blue: DE with Vertical load, Red: SE with Vertical load, Green: DE with Oblique load, and Purple: SE with Oblique load).

The principal stress and displacement of the adhesive layer of the SE RBFPDs under oblique loading were the largest in all test groups, as shown in Figure 7, and was the same as the principal stress of the restoration.

### Stress of enamel

**FIGURE 8:**
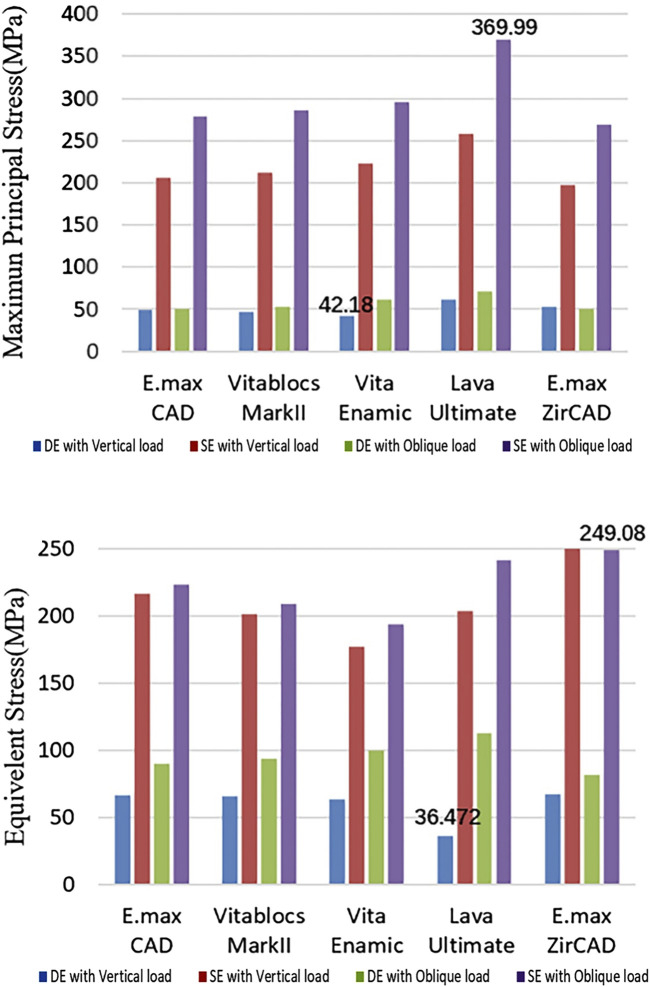
Quantification of the stress of enamel (Blue: DE with Vertical load, Red: SE with Vertical load, Green: DE with Oblique load, and Purple: SE with Oblique load).

The enamel stress of the Lava Ultimate SE RBFPDs was the highest in all test groups under oblique loading, like the principal stress of the restoration, as shown in Figure 8. The equivalent stress value of the enamel layer of IPS e.max ZirCAD SE RBFPDs was the highest under oblique loading, whereas that of Lava Ultimate DE RBFPDs was the smallest. No significant differences were observed in stress or displacement between SE and DE RBFPDs under oblique loading.

### Failure probability

**FIGURE 9:**
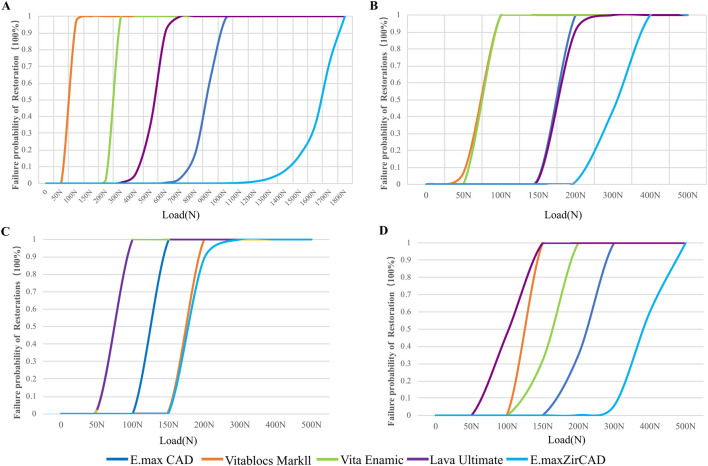
Failure probability of restorations according to the Weibull risk-of-failure analysis for **(A)** double-ended model with vertical load, **(B)** single-ended model with vertical load, **(C)** double-ended model with oblique load and **(D)** single-ended model with oblique load.

**FIGURE 10:**
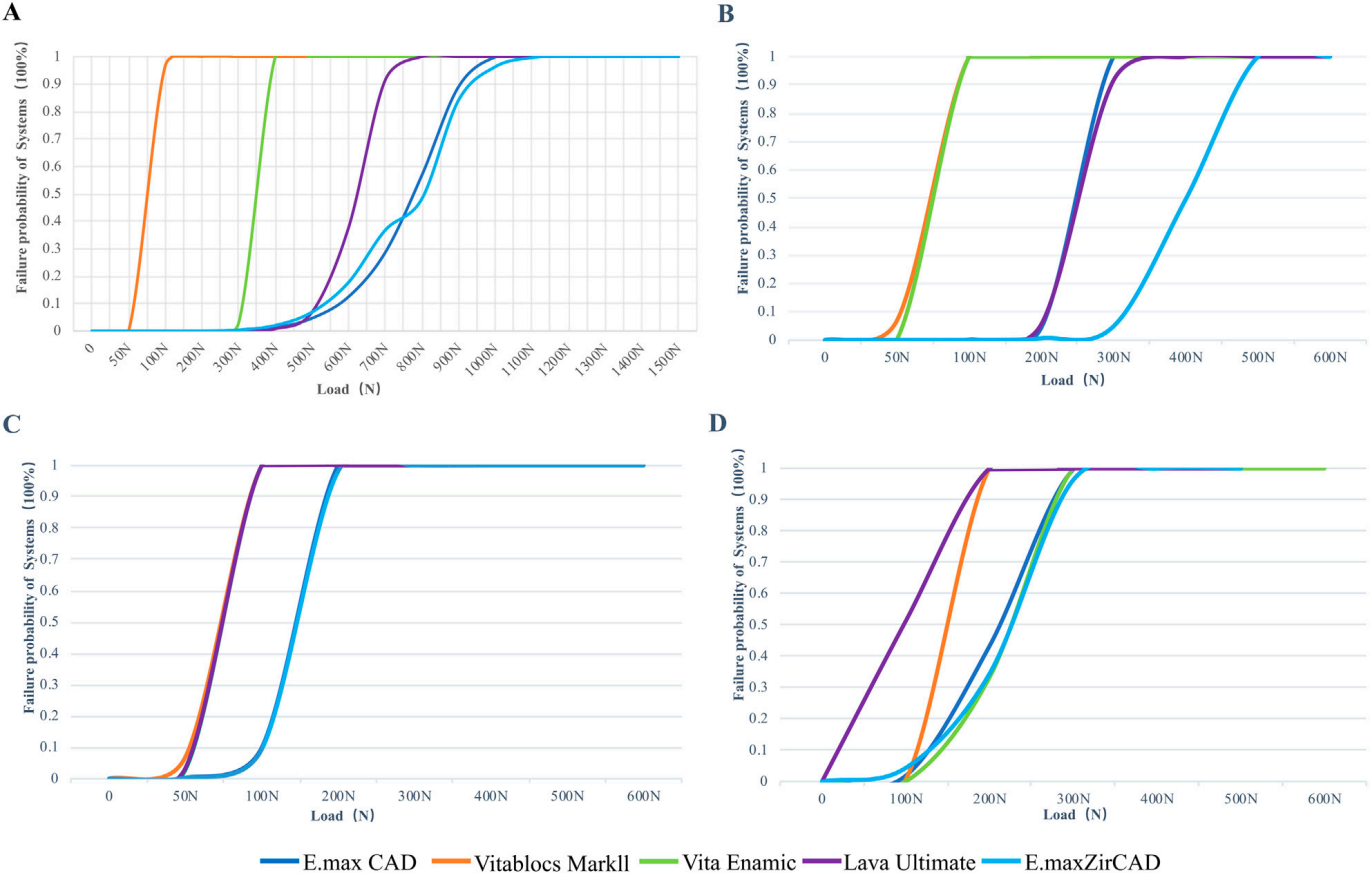
Figure 9: Failure probability of systems according to the Weibull risk-of-failure analysis for **(A)** double-ended model with vertical load, **(B)** single-ended model with vertical load, **(C)** double-ended model with oblique load and **(D)** single-ended model with oblique load.

The failure probability of the RBFPDs restorations was significantly different between materials, as shown in Figure 9. The failure probability of Vita Enamic was the highest, that of IPS e.max ZirCAD was the lowest, and that of IPS e.max CAD was similar to that of IPS e.max ZirCAD. Oblique force shows a higher probability of failure than vertical force regardless of the type of material (SE rather than DE), as shown in Figure 10. The failure probability of Lava Ultimate in the SE model was higher than that of the DE model, and that of the IPS e.max ZirCAD DE RBFPDs was the lowest when it was vertically stressed. Therefore, both null assumptions tested on the present study were not valid.

## Discussion

In recent years, innovative CAD/CAM technology has added a new dimension to repairing missing anterior teeth with all-ceramic RBFPDs beside the chair. The choice of the CAD/CAM material and repair design is a problem worth considering. Therefore, this study used FEA and Weibull analyses to evaluate the effects of different CAD/CAM materials and restoration designs on the stress distribution and failure of anterior teeth under adhesive bridge restoration to provide a reference for clinicians in mathematical simulation biomechanics.

FEA is becoming increasingly popular in dental research because it contributes to the study of complex geometries and the influence of the geometry or strength of different materials (Motta et al., 2014). The stress distribution of each component can be predicted by simulating the occlusal load condition in a finite-element model (Duan and Griggs, 2015). The influence of the material or repair design on the biomechanical properties of the adhesive bridge can be revealed. However, despite its advantages, FEA has several limitations when compared to clinical reality. The simplifications made during model creation, such as the assumptions of boundary conditions, the approximations of material properties, and the geometric simplifications, might introduce discrepancies between the FEA model and actual biomechanical responses. For instance, in practice, dental structures and tissues often exhibit nonlinear, anisotropic behaviors that are difficult to capture through idealized FEA models. Similarly, individual patient differences, such as variations in mechanical properties of materials, tooth morphology, and bone biomechanics, are challenging to model accurately. Nonetheless, FEA provides a valuable tool for stress and failure analysis under controlled conditions, especially when combined with more detailed failure risk models. FEA cannot fully predict the risk and lifespan of dental restoration failures on its own. This requires Weibull analysis (Sato et al., 1995), which can calculate the fracture probability of brittle materials and predict the cumulative failure probability under different stress levels (Arat Bilhan et al., 2015). By integrating both FEA and Weibull analysis, we can obtain a more comprehensive understanding of the biomechanical performance of different materials and designs, offering insights that bridge the gap between computational models and clinical practice.

Materials with a lower elastic modulus are generally more flexible and less rigid, resulting in greater deformation under applied stress, but they are less prone to fracture under smaller loads. For instance, Lava Ultimate, characterized by its relatively low elastic modulus, exhibits significant flexibility, which may aid in stress absorption in low-stress areas. However, this flexibility can increase the risk of failure in high-stress regions due to localized stress concentration. Conversely, materials with a higher elastic modulus, such as IPS e.max ZirCAD, display superior rigidity, enabling more uniform stress distribution and reducing the likelihood of localized failure under high-load conditions. In the context of resin-bonded bridges, it is crucial to consider not only the rigidity of the material but also its bonding performance, as resin-bonded bridges rely heavily on strong adhesive properties to withstand long-term functional stresses. Furthermore, materials with a lower Poisson’s ratio, such as Vitablocs MarkII, exhibit minimal lateral deformation under vertical loads, which enhances stability and reduces the risk of bonding failure. On the other hand, materials with a higher Poisson’s ratio, like Lava Ultimate, tend to undergo greater lateral deformation, particularly under oblique loading, which can increase shear stress at the bonding interface. This may result in stress concentration and elevate the risk of adhesive failure. Therefore, in the selection of materials for resin-bonded bridges, those with a lower Poisson’s ratio are generally preferable, as they mitigate the impact of lateral deformation and shear stress, potentially improving the long-term performance and durability of the restoration.

In the stress distribution map (Figure 2), it is observed that the SE bridge exhibits stress concentration primarily on the abutment teeth, while the DE bridge shows stress concentrated at the incisal edge of the bridge and the adjacent areas of the abutment teeth. In contrast, the periodontal membrane experiences relatively low stress. The periodontal membrane, being a viscoelastic soft tissue, can deform when subjected to occlusal or other forces, acting as a cushion to transfer pressure from the teeth to the surrounding bone. Due to the relatively high elastic modulus of the periodontal membrane, the applied 100 N occlusal force is within the tolerance range of the periodontal membrane, resulting in stress concentration at the bonding interface between the abutment teeth and the wing plates. Consequently, the primary failure mode of the restoration is detachment of the prosthesis, with minimal impact on the periodontal condition of the abutment teeth. When a denture performs its function, it is exposed to breakage. A phenomenon of stress concentration between the bridge body and restoration when loading on the bridge body has been previously described (Lundgren and Laurell, 1986a; Lundgren and Laurell, 1986b). The stress value of RBFPDs is higher, and the deformation of the wing arm is large. This is consistent with the experimental results showing that the maximum principal stress of SE RBFDPs is concentrated on the connector’s palatal side near the incisal end. However, that of DE RBFDPs is located on the labial side near the neck. According to material mechanics analysis, the self-resistance of RBFPDs can be improved by increasing the joint area’s thickness, width, and obtuse angle. This suggests that when repairing the lower anterior teeth, the connector’s area and wing plate thickness should be increased as much as possible to increase the strength of the RBFPD without affecting the abduction gap’s physiological function. The concave depth of the teeth near the gap of the adjacent teeth can be adjusted slightly to increase the thickness of the RBFPD. In the clinical application of SE RBFPDs, it is beneficial to increase the connector thickness.

In this study, the maximum principal stress and displacement of DE RBFPDs were lower than those of SE cantilever RBFPDs, which is consistent with previous research (Rosentritt et al., 2009b). Weibull analysis also shows that the failure probability of an SE RBFPD is higher than that of a DE one. However, does this mean that DE RBFPDs are better than SE RBFPDs? Some studies have shown that SE all-ceramic RBFPDs are more widely used in clinics and have higher success rates (Botelho et al., 2014). SE RBFPDs of anterior teeth are the most promising for permanent restoration. When the bonding bridge is under stress, fatigue caused by the complex stress caused by the inconsistency in abutment mobility is the main cause of bond failure. In clinical practice, it is also found that SE RBFPDs have a high survival rate, mainly because there is no difference in abutment mobility between SE RBFPDs. When there is a significant difference in the mobility of the abutment teeth, DE RBFPDs are affected by complex stresses, resulting in bond failure. Therefore, in accordance with the bond area and periodontal support theories, the lack of contralateral incisors can also be the first choice for designing SE RBFPDs. Sailer et al. showed that the 6-year cumulative survival rate of anterior teeth SE all-ceramic RBFPDs was 100%, and the shedding rate was 56% (Sailer et al., 2013). In another study with an average service life of 10 years, the cumulative survival rate of all-ceramic DE RBFPDs was 73.9%, and that of SE all-ceramic RBFPDs was 94.4% (Kern and Sasse, 2011b). While DE RBFPDs exhibit lower overall stress, SE RBFPDs can perform better in certain biomechanical situations due to their simpler design and stress distribution. With only one abutment, SE RBFPDs avoid the complications associated with differential mobility between two abutments, which is a common issue in DE RBFPDs. This differential movement can lead to bond failure when one abutment experiences more movement than the other under load. Additionally, SE RBFPDs tend to have a more predictable stress distribution, reducing the risk of failure caused by high-stress concentration in both abutments. As such, in cases where mobility is more predictable, especially in anterior teeth where forces are generally lower, SE RBFPDs often provide better biomechanical performance by avoiding complications related to differential loading. However, in all cases, SE RBFPDs is not superior to DE RBFPDs. The DE design should be considered for teeth with a larger occlusal force, such as posterior and lower anterior teeth with smaller enamel bonding surfaces. In addition, only the DE design can achieve the desired clinical effect when it is used as a periodontal splint or fixed retainer after orthodontic treatment.

In this experiment, it is suggested that the choice of CAD/CAM material is very important for the failure probability of RBFPDs. Among them, the failure probability of Vita Enamic was the highest, and that of IPS e.max ZirCAD was the lowest. No significant difference was observed between IPS e.max CAD and IPS e.max ZirCAD. This conclusion is consistent with previous reports. The 3-year success rate of anterior tooth IPS hot-pressing casting all-ceramic RBFPDs was 88.5%, and the 2-year cumulative survival rate of zirconia all-ceramic RBFPDs was 90% (Zhou et al., 2011; Zhou et al., 2010). The 5-year follow-up results of Sasse et al. showed that the cumulative survival rate of zirconia all-ceramic RBFPDs is 100% (Sasse and Kern, 2013). FEA and Weibull analysis showed that oblique loading had a higher failure probability than vertical loading, regardless of the type of material. The bonding surface of RBFPDs is also affected by multidirectional forces, and horizontal force is more destructive to the bonding interface than vertical force. Therefore, it is recommended that in the design of RBFPDs, it is best to require patients with shallow overlay, shallow coverage, and only slight occlusal contact in apical dislocation and lateral and protruding movements.

## Conclusion and future work

The finite element analysis (FEA) and Weibull analysis provided several key insights. The stress distribution in both single-ended (SE) and double-ended (DE) resin-bonded fixed partial dentures (RBFPDs) revealed that the connector areas experience the largest stresses. Notably, the maximum principal stress and displacement in DE RBFPDs were lower than those in SE RBFPDs. This indicates that DE designs may provide better stress distribution and mechanical stability.

Additionally, the failure probability under oblique reinforcement was higher compared to vertical reinforcement, particularly in DE RBFPDs. This highlights the increased vulnerability of RBFPDs under non-vertical forces, which should be carefully considered in clinical applications.

When comparing materials, Vita Enamic exhibited the highest failure probability, while IPS e.max ZirCAD had the lowest. There was no significant difference in failure probability between IPS e.max CAD and IPS e.max ZirCAD, suggesting that both materials are suitable choices with comparable performance under tested conditions.

This study utilized complex and accurate finite element modeling based on cone-beam computed tomography (CBCT) data, offering a deeper understanding of the stress distribution and failure probabilities in adhesive bridges. The results provide valuable biomechanical insights for selecting restoration designs and CAD/CAM materials for lower anterior teeth adhesive bridges.

Future work will focus on incorporating dynamic loading conditions to better simulate the complex masticatory movements in the oral cavity. Additionally, efforts will be made to model the heterogeneity and viscoelasticity of biological tissues more accurately, which will enhance the clinical relevance and accuracy of these analyses.

## Data Availability

The raw data supporting the conclusions of this article will be made available by the authors, without undue reservation.
